# Genetic testing for familial epilepsies: Diagnostic yield and genetic findings

**DOI:** 10.1002/epi.70160

**Published:** 2026-03-08

**Authors:** Colin A. Ellis, Juliette Copeland, Isabella Velez, Karen L. Oliver, Hannah Shalaby, Aaron Baldwin, Caren Armstrong, Amanda Back, Brianna Berlin, Stacey Cohen, Vishnu Anand Cuddapah, Danielle deCampo, Holly Dubbs, Natalie Ginn, Alicia G. Harrison, Naomi Lewin, Laina Lusk, Eric D. Marsh, Shavonne L. Massey, Pamela Pojomovsky McDonnell, Jillian L. McKee, Xilma Ortiz‐Gonzalez, Anna J. Prentice, Katie Rose Sullivan, Sarah M. Ruggiero, Mark P. Fitzgerald, Ethan M. Goldberg, Ingo Helbig

**Affiliations:** ^1^ Department of Neurology University of Pennsylvania Philadelphia Pennsylvania USA; ^2^ Epilepsy Neurogenetics Initiative (ENGIN) Children's Hospital of Philadelphia Philadelphia Pennsylvania USA; ^3^ Drexel University College of Medicine Philadelphia Pennsylvania USA; ^4^ Epilepsy Research Centre, Department of Medicine University of Melbourne, Austin Health Heidelberg Victoria Australia; ^5^ Genetics and Gene Regulation Division The Walter and Eliza Hall Institute of Medical Research Parkville Victoria Australia; ^6^ Division of Neurology Children's Hospital of Philadelphia Philadelphia Pennsylvania USA; ^7^ Department of Biomedical and Health Informatics Children's Hospital of Philadelphia Philadelphia Pennsylvania USA; ^8^ Department of Neuroscience University of Pennsylvania Philadelphia Pennsylvania USA; ^9^ Present address: Department of Neurology University of California Davis Davis California USA; ^10^ Present address: Department of Human Genetics University of Pittsburgh, School of Public Health Pittsburgh Pennsylvania USA; ^11^ Present address: Jan and Dan Duncan Neurological Research Institute, Texas Children's Hospital Houston Texas USA; ^12^ Present address: Division of Pediatric Neurology and Developmental Neuroscience, Department of Pediatrics Baylor College of Medicine Houston Texas USA

**Keywords:** exome sequencing, familial epilepsy, family history, gene panel, genetic testing

## Abstract

**Objective:**

Genetic testing has become a routine part of clinical epilepsy care. Family history is an indication for genetic testing, but the diagnostic yield, predictors of a genetic diagnosis, and association with familial patterns are not well understood.

**Methods:**

This was a retrospective cohort study of genetic testing performed at pediatric and adult epilepsy genetics clinics. Eligible patients (probands) had epilepsy and one or more first‐degree relatives or two or more other relatives with epilepsy. Genetic testing strategies were patient specific, reflecting real‐world clinical practice. Familial patterns were classified based on affected relatives of the proband. Diagnostic variants were tested in the proband's parents when possible.

**Results:**

We studied 484 probands and their families. A genetic diagnosis was identified in 99 of 484 (20%). Predictors of a genetic diagnosis were presence of neurodevelopmental disorder (*X* ^2^(1) = 9.6, *p* = .002) and earlier age at seizure onset (Mann–Whitney *U* test, *p* < .001). The likelihood of a genetic diagnosis was not associated with epilepsy type, drug resistance, brain magnetic resonance imaging (MRI) findings, number of affected first‐degree relatives, total number of affected relatives, or having an affected parent with epilepsy. Among those with genetic diagnoses, variant segregation matched the familial pattern of affected individuals in 79%. The other 21% of families had unexpected segregation, including de novo variants in patients with affected ancestors and inherited variants in patients with no known affected ancestors.

**Significance:**

Familial epilepsy has a substantial rate of genetic diagnosis and is an appropriate indication for genetic testing. Pedigree‐related factors did not influence the likelihood of genetic diagnosis, suggesting that all families can be considered for genetic testing, independent of inheritance patterns and number of affected relatives. Familial patterns can help interpret genetic test results, while also revealing the complexities of incomplete penetrance and independent epilepsy etiologies in families.

**Key points**

- A genetic diagnosis was identified in 99 of 484 (20%) individuals with epilepsy and a family history of epilepsy.
- Familial epilepsy is an appropriate indication for genetic testing.
- Pedigree‐related factors did not influence the likelihood of genetic diagnosis; all families can be considered for genetic testing.
- Familial patterns can help interpret genetic test results, but can also be misleading.

## 1 INTRODUCTION

Decades of studies of families and twins indicate that epilepsy has strong genetic influences.^1^, ^2^ Families with multiple individuals affected with epilepsy are common: among people diagnosed with epilepsy, 10–20% have a positive family history of epilepsy.^3^, ^4^, ^5^ A positive family history of epilepsy may prompt clinicians to consider genetic etiologies, and genetic testing. Professional society recommendations include family history and familial epilepsies as important indications for genetic testing in clinical practice.^6^, ^7^ However, few data exist on how these recommendations are implemented in clinical care.

The past decade has seen tremendous progress in discovery of causative genes for epilepsy, with now over 1000 identified epilepsy‐associated genes.^8^, ^9^ Clinical genetic testing in appropriately selected patients has an overall diagnostic yield of 15%–25%,^10^, ^11^, ^12^ and even higher in specific epilepsy subtypes.^6^ However, most studies of genetic testing for epilepsy have focused on individuals with developmental and epileptic encephalopathies, or other neurodevelopmental disorders, leaving other clinically relevant subgroups less well characterized.

Although family history of epilepsy is a common reason for genetic testing, the outcomes of real‐world clinical genetic testing for familial epilepsies has not been systematically studied. In the early days of epilepsy gene discovery, recognizable familial syndromes such as genetic epilepsy with febrile seizures plus (GEFS+), autosomal dominant sleep‐related hypermotor epilepsy (ADSHE), and autosomal dominant epilepsy with auditory features (ADEAF) led to the initial gene discoveries in the field.^6^, ^13^ More recently, gene discovery has shifted to focus on epileptic encephalopathies and other neurodevelopmental disorders caused by de novo variants, with less attention to families and familial epilepsies. Special familial syndromes are relatively rare, and little is known about the broader population with a family history of epilepsy. A recent meta‐analysis of 154 studies of genetic testing for epilepsy did not include analysis of family history, noting that it was not routinely reported in the publications.^10^ One study described genetic etiologies of 211 Israeli and Palestinian families with two or more relatives with epilepsy, and identified pathogenic variants in 23% of families.^14^ However, these families were ascertained for research‐based genetic analysis, had their genetics tested using a variety of research techniques, and contributed to 11 new gene discoveries. These factors may limit the generalizability of the findings to current clinical practice in other populations.

In this study, we analyzed the clinical genetic testing results of individuals with familial epilepsy, defined as the patient (proband) plus at least one first‐degree relative or two second‐ or third‐degree relatives with epilepsy. The cohort includes patients across the lifespan from pediatric to adult care across two academic epilepsy centers, and reflects real‐world clinical practice, with dynamic genetic testing strategies and multiple testing modalities.

## 2 METHODS

### 2.1 Cohort selection and data collection

This was a retrospective cohort study of consecutive patients seen in the epilepsy genetics clinic at the Children's Hospital of Philadelphia (CHOP) and Penn Medicine, with institutional review board (IRB) approval at each institution. These clinics perform genetic testing and counseling for epilepsy and account for the vast majority of clinical genetic testing for epilepsy at our institutions. The clinics are staffed by physicians and genetic counselors with expertise in epilepsy genetics, genetic diagnosis, and variant interpretation. The clinics maintain prospective databases of all patients seen and genetic testing performed, and phenotype data including family history. This study used data from 2/1/2016 to 7/1/2025 (Penn) and 1/1/2019 to 7/1/2025 (CHOP).

This study included patients with familial epilepsy, defined as the proband plus at least one first‐degree relative or two second‐ or third‐degree relatives with epilepsy. Each family had exactly one designated proband—for example, if two siblings were evaluated together, one was designated the proband and the other as an affected sibling. We did not include individuals with febrile seizures, provoked seizures, a single unprovoked seizure, or known acquired causes of epilepsy. For consistency we only considered relatives within a three‐generation pedigree for each proband (i.e., siblings, parents, grandparents, aunts and uncles, and cousins, plus the children of adult probands) because more distant relatives are inconsistently known or reported. The documented family history systematically captured affected relatives, but did not systematically capture unaffected relatives, and complete pedigrees were not available for all subjects, so our analysis was limited to known affected individuals within families. We excluded probands who were seen for genetic confirmation of a known pathogenic variant in the family. We excluded probands who had genetic tests pending at the end of the study period. Phenotype data collected for probands include age, sex, epilepsy type, age at seizure onset, presence of intellectual disability, and brain magnetic resonance imaging (MRI) findings. Data missing from the clinical databases were collected through chart review.

### 2.2 Genetic testing

Genetic testing was performed clinically, at the discretion of the epilepsy genetics clinic teams. This analysis includes chromosomal microarray, epilepsy gene panels, and exome sequencing, which are the most common testing modalities. Genome sequencing was completed on only three eligible participants, and so for this analysis, is combined with exome sequencing. Variants were interpreted at the time of testing using standard criteria of the American College of Medical Genetics and Genomics (ACMG),^15^ and evaluated in clinical context by the epilepsy genetics clinic team. A genetic diagnosis was defined as a pathogenic or likely pathogenic variant in a gene with dominant inheritance, or biallelic pathogenic or likely pathogenic variants in a gene with recessive inheritance, compatible with the patient's phenotype. In some cases, variants of uncertain significance were interpreted as diagnostic by the epilepsy genetics team, based on characteristics of the variant and the patient's phenotype. In those cases, the classification used in this analysis reflects the final interpretation of the epilepsy genetics clinical team. When possible, probands' parents were tested for variant segregation. We did not systematically test relatives other than parents, and they are not included in this analysis. We did not collect data on nondiagnostic results (e.g., variants of uncertain significance that were not considered diagnostic), carrier status of heterozygous pathogenic variants in recessive genes, or secondary findings unrelated to the patient's epilepsy. We did not distinguish exome initial analysis from reanalysis.

### 2.3 Frequency of genetic etiologies in all epilepsies undergoing genetic testing

We sought to qualitatively compare the distribution of genetic etiologies in our familial cohort against the broader population of people with epilepsy undergoing genetic testing, without specific selection for family history. To construct the latter distribution we pooled the results of the five largest published case series to date on the diagnostic yield of genetic testing for epilepsy.^11^, ^12^, ^16^, ^17^, ^18^ Collectively these studies collectively included 22 854 individuals from three continents. Family history of epilepsy was 14% and 34% in the two studies that reported it.^16^, ^18^ For each study we calculated the proportion of each genetic etiology, and then we averaged these proportions between studies so as to give equal weight to each study. If a given gene was not tested in a study it did not contribute to the average.

### 2.4 Familial patterns and variant segregation analysis

**FIGURE 1:**
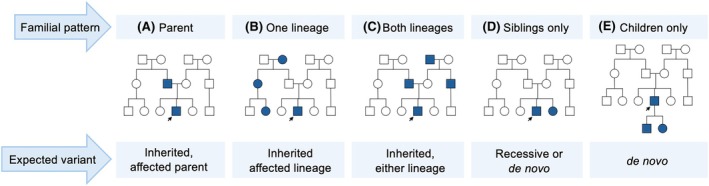
Familial patterns and expected variant segregation. We classified familial patterns based on affected relatives of the proband (top row), and designated the expected results of parental variant testing for each familial pattern (bottom row). (A) One parent of proband affected; expect that variant was inherited from affected parent. (B) Affected ancestors of proband in one parental lineage; expect that variant was inherited from the parent in the affected lineage. (C) Affected ancestors of proband in both parental lineages; expect that variant inherited from either parent. (D) Only siblings affected, with no affected ancestors; expect biallelic recessive variants, or de novo with parental mosaicism (detected or presumed). (E) Only children affected, with no affected ancestors to proband; expect de novo variant in the proband. In the pedigrees, squares represent males, circles represent females, filled symbols represent individuals affected with epilepsy, unfilled symbols represent unaffected individuals, and small arrowheads indicate the proband, that is, the patient undergoing genetic testing.

When a proband had potentially diagnostic variant(s), parental testing was performed, if possible, for variant segregation. We classified familial patterns based on affected relatives of the proband, and designated the expected parental segregation of diagnostic variants as follows (Figure 1):

- Group A: One parent of proband affected: expect variant inherited from affected parent.
- Group B: Affected ancestors of proband in one parental lineage: expect variant inherited from the parent in the affected lineage.
- Group C: Affected ancestors of proband in both parental lineages: expect variant inherited from either parent.
- Group D: Only siblings affected, with no affected ancestors: expect biallelic recessive variants, or de novo with parental mosaicism (detected or inferred).
- Group E: Only children affected, with no affected ancestors to proband: expect de novo variant in the proband.

Note that Groups A and B are applicable to autosomal dominant, X‐linked, or mitochondrial modes of inheritance. That is, the grouping system here is not specific to modes of inheritance; rather, the concept is that affected ancestors are compatible with an inherited causal variant, whereas no affected ancestors are compatible with recessive or de novo variants.

### 2.5 Statistical analysis

The unit of analysis was the proband of each family. For univariable associations between clinical variables and likelihood of a genetic diagnosis, we performed chi‐square tests or Fisher exact for categorical variables, Cochran–Armitage trend tests for ordinal variables, and non‐parametric Mann–Whitney *U* tests for continuous variables.

## 3 RESULTS

### 3.1 Cohort characteristics

A total of 519 probands met inclusion criteria, of whom 484 (93%) completed the recommended genetic testing and were included in this analysis. Cohort characteristics are shown in Table 1. The median number of affected relatives per proband was 2 (range 1–6). The CHOP clinic contributed 384 pediatric cases and Penn contributed 100 adult cases.

**TABLE 1:** Cohort characteristics.

Characteristic	Cohort of 484 probands
Sex female, *n* (%)	247 (51%)
Age at evaluation, median (IQR)	11 (5, 17)
Age at epilepsy onset, median (IQR)	5 (2, 11)
Drug resistant, *n* (%)	124 (26%)
Intellectual disability, *n* (%)	101 (21%)
Epilepsy type, *n* (%)	
Focal	209 (43%)
Generalized	138 (29%)
DEE	46 (10%)
Febrile seizures plus	11 (2%)
Unclassified	63 (13%)
Brain MRI, *n* (%)	
Normal or nonspecific	393 (75%)
Malformation of cortical development	31 (6%)
Other epileptogenic lesion	22 (5%)
MRI not performed/not available	68 (14%)

Abbreviations: DEE, developmental and epileptic encephalopathy; IQR, interquartile range; MRI, magnetic resonance imaging.

Thirty‐five eligible probands did not complete testing due to insurance denial (*n* = 26) or patient/family declined (*n* = 9). These 35 probands had characteristics similar to that of the 484 who completed testing and were included in the analysis (Table S1).

### 3.2 Diagnostic yield and genetic diagnoses

**FIGURE 2:**
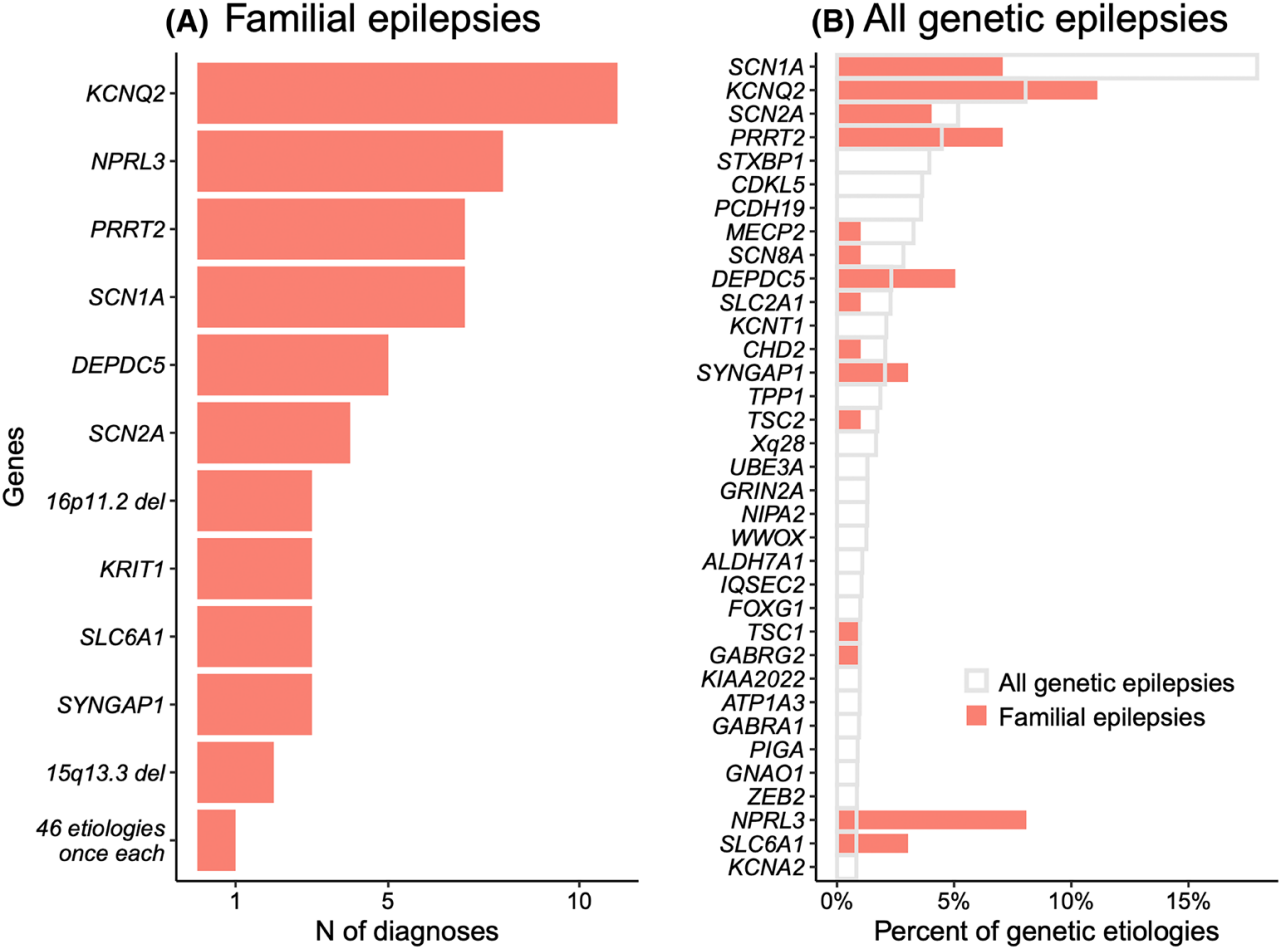
Distribution of genetic etiologies. (A) The cohort included 57 unique genetic etiologies, of which 11 were present more than once and 46 were present once. (B) We compared the distribution of genetic etiologies in our familial cohort (red) against the distribution of genes in the broader population of people with epilepsy undergoing genetic testing (white) based on pooled results of the five largest published case series to date on the diagnostic yield of genetic testing for epilepsy [refs ^11^, ^12^, ^16^, ^17^, ^18^].

A genetic diagnosis was identified in 99 of 484 families (20%). These included 57 unique genetic etiologies, of which 11 were present more than once and 46 were present only once (Figure 2). Mode of inheritance of the 57 etiologies was autosomal dominant (*n* = 44), autosomal recessive (6), X‐linked (4), mitochondrial (2), and trisomy (1). Of the six families with autosomal recessive diagnoses, five were compound heterozygous and one was homozygous in a family with known consanguinity. Thirteen patients had diagnostic copy number variants. A complete list of genetic diagnoses is in Table S2.

### 3.3 Clinical predictors of diagnostic yield

**FIGURE 3:**
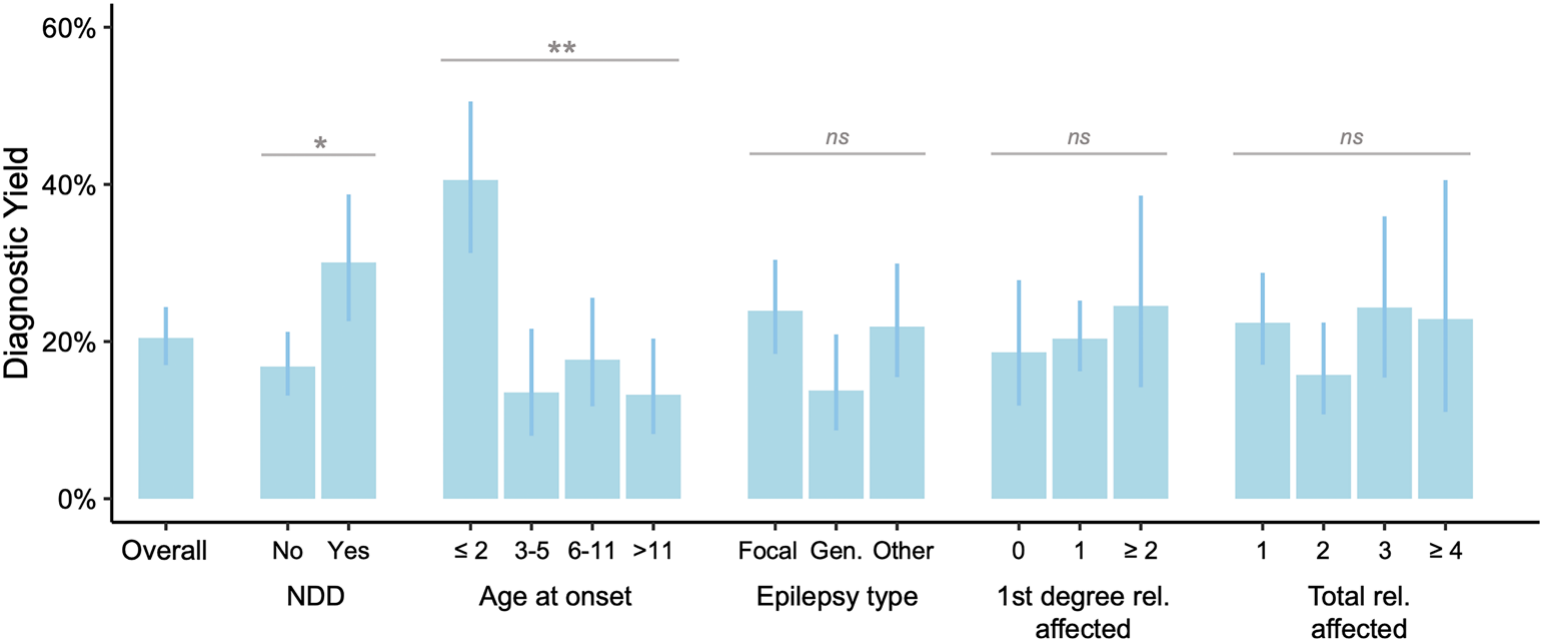
Diagnostic yield of genetic testing in familial epilepsies. The diagnostic yield (diagnostic tests divided by tests performed) for the cohort overall, and stratified by subgroups based on presence of neurodevelopmental disability, age at seizure onset, epilepsy type, number of affected first‐degree relatives, and total number of affected relatives. Gen., generalized; NDD, neurodevelopment disability; Rel., relatives. Significance testing: *p < .01, **p < .001.

Predictors of a genetic diagnosis (Figure 3) were presence of neurodevelopmental disorder (*X* ^2^(1) = 9.6, *p* = .002) and earlier age at seizure onset (Mann–Whitney *U* test, *p* < .001). However, the absence of these predictors did not preclude a genetic diagnosis: among 120 probands without neurodevelopmental disorder and age at seizure onset >11 years, a genetic diagnosis was identified in 14 of 120 (12%).

The likelihood of a genetic diagnosis was not associated with the following factors: epilepsy type (Fisher exact *p* = .12), number of affected first‐degree relatives (Cochran–Armitage trend test, *X* ^2^(1) = 1.0, *p* = .31), total number of affected relatives (*X* ^2^(1) = .02, *p* = .88), drug‐resistant epilepsy (*X* ^2^(1) = 1.8, *p* = .18), brain MRI finding (Fisher exact *p* = .91), or having an affected parent with epilepsy (*X* ^2^(1) = 1.9, *p* = .17). There was no difference in diagnostic rate between the pediatric and adult centers (CHOP 21%, Penn 17%, *X* ^2^(1) = .68, *p* = .41).

### 3.4 Genetic tests

Genetic tests performed are shown in Table 2. A proband could have more than one genetic test. Of the 484 probands, 365 individuals (75%) had one genetic test, 101 (21%) had two tests, and 18 (4%) had three tests (Table S3). Three hundred eighty‐three probands (79%) had sequencing tests only (gene panel, exome or genome sequencing), 15 probands (3%) had analysis of copy number variants only, and 86 probands (18%) had both sequencing and copy number analysis. Genome sequencing was performed in three patients, of which two were diagnostic. Both diagnoses were exonic variants that, in theory, could have been detected by exome sequencing or gene panel analysis.

**TABLE 2:** Genetic tests performed.

Genetic test	Performed (N)	Diagnostic (N)	Yield (%)
Chromosomal microarray	101	9	9%
Gene panel	326	53	16%
Exome or genome sequencing	193	34	18%

### 3.5 Familial patterns and segregation analysis

**FIGURE 4:**
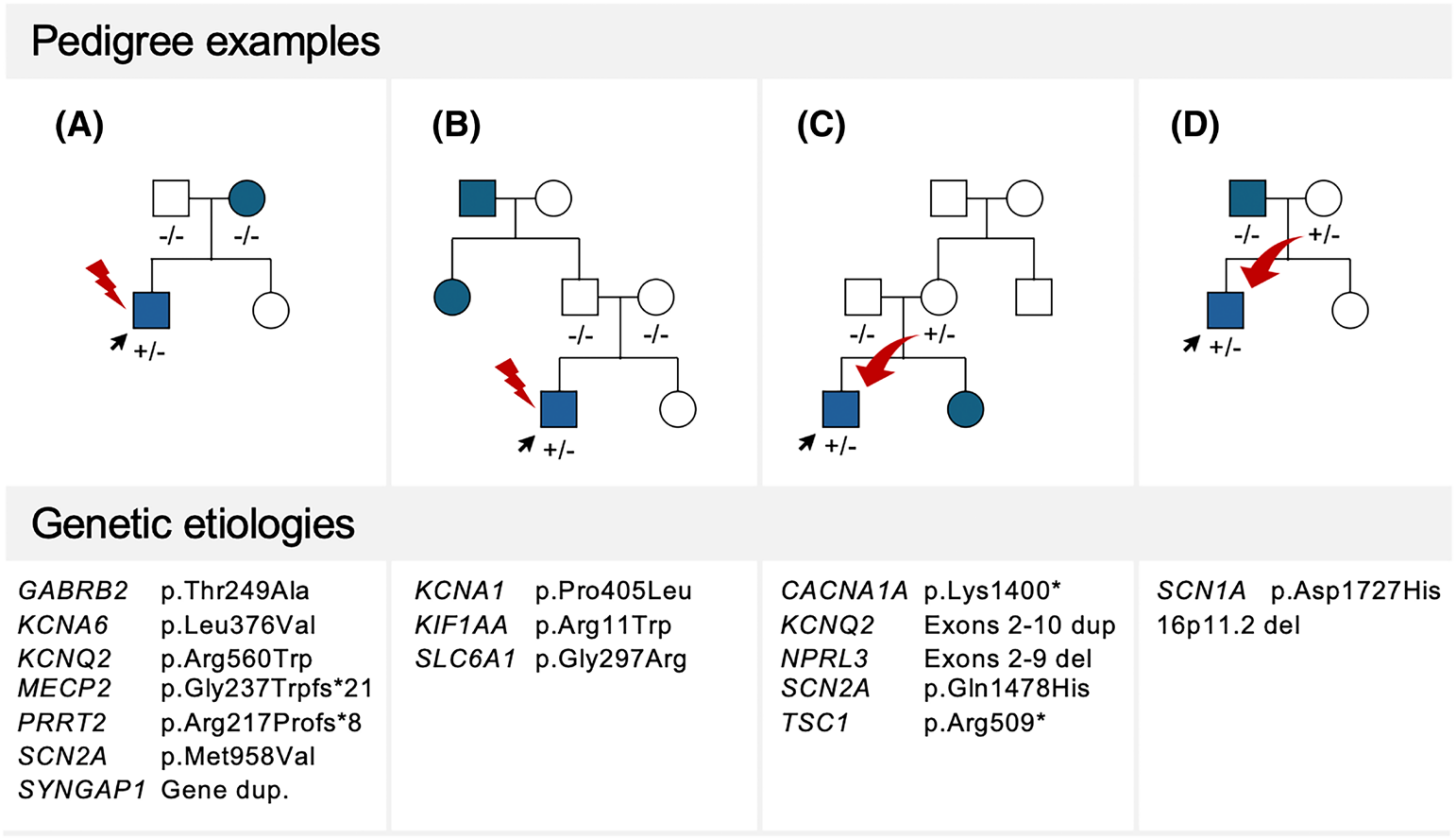
Examples of unexpected segregation. Unexpected segregations included (A) diagnostic de novo variants in patients with an affected parent, in seven families; (B) diagnostic de novo variants in patients with affected ancestors, in three families; (C) dominantly inherited diagnostic variants in patients with no affected ancestors, in five families; and (D) dominantly inherited variants from an unaffected parent while the other (non‐transmitting) parent was affected. In the pedigrees, squares represent males, circles represent females, filled symbols represent individuals affected with epilepsy, unfilled symbols represent unaffected individuals, plus signs (+) indicate the diagnostic variant, minus signs (‐) indicate absence of the variant, red arrows represent transmission of the variant from parent to offspring, and red thunderbolts represent de novo variants.

Among the 99 genetic diagnoses, 81 had parental testing allowing variant segregation. Variant segregation matched the familial pattern in 64 of 81 families (79%). The remaining 17 of 81 families (21%) had unexpected segregation. Examples of unexpected segregations are shown in Figure 4. Diagnostic de novo variants were identified in 10 patients with affected ancestors, including affected parents in 7 cases. Four additional families with diagnostic de novo variants had only affected siblings, indicating parental germline mosaicism, classified as expected segregation. Five probands inherited their diagnostic variant from an unaffected parent and had no known affected ancestors, only affected siblings or children (genes *KCNQ2*, *NPRL3*, *TSC1*, *SCN2A*, and *CACNA1A*). Two probands each had one affected parent but inherited their diagnostic variants from the unaffected parent (*SCN1A* and 16p11.2 deletion). The diagnostic yield stratified by familial pattern groups is shown in Table S4.

## 4 DISCUSSION

Here we report a retrospective study of clinical genetic testing for 484 patients with familial epilepsy, defined as the patient plus at least one first‐degree relative or two second‐ or third‐degree relatives with epilepsy. Across the entire cohort, the diagnostic yield of genetic testing was 20%. The presence of neurodevelopmental disability and earliest age at seizure onset were associated with higher likelihood of a genetic diagnosis. In contrast, familial factors such as number of affected relatives, affected first‐degree relatives, and having an affected parent were not associated with higher likelihood of genetic diagnosis. Parental testing of diagnostic variants showed the expected segregation pattern in 79% of families, and unexpected segregation in 21%.

There are several main insights from our study. First, our findings support the guidance that family history is an appropriate indication for genetic testing.^6^, ^7^ Diagnostic yield remained relatively high (≥12%), even in the absence of neurodevelopmental disability and early age at onset, which are well‐established predictors of a genetic diagnosis in non‐familial epilepsy cohorts.^6^, ^10^ Second, within familial epilepsies, we did not identify a familial feature that strongly predicted a genetic diagnosis. This observation contrasts the traditional notion of monogenic familial epilepsy syndromes such as genetic epilepsy with febrile seizures plus (or GEFS+), autosomal dominant sleep‐related hypermotor epilepsy (or ADSHE), or autosomal dominant epilepsy with auditory features (ADEAF), which produced the first major gene discoveries in the field. This suggests that genetic testing can be considered for any family meeting a broad definition of familial epilepsy, such as the definition we used here: the proband plus at least one first‐degree relative or two second‐ or third‐degree relatives with epilepsy.

Third, although there is substantial literature on the diagnostic yield of clinical genetic testing in patients with epilepsy, no previous study has focused specifically on familial epilepsies. Several prior studies reported diagnostic yield in the subset of a larger cohort who had a positive family history (Table S5).^18^, ^19^, ^20^, ^21^, ^22^, ^23^, ^24^, ^25^, ^26^, ^27^, ^28^ These subcohorts were small (all <100 cases, most <50), varied in their definitions of family history, and none performed further analysis of the familial cohorts. Our study builds on this prior work by systematically studying a large cohort of familial epilepsies, exploring for the first time the familial factors and segregation patterns associated with genetic diagnoses in these families.

Afawi et al.^14^ described genetic analysis of 211 Israeli and Palestinian familial epilepsies. They identified pathogenic variants in 49 of 211 families (23%). Genetic analysis was performed using research methods, such as targeted sequencing of known and candidate genes. Ascertainment was not systematic, and some families were part of the initial discovery cohorts for 11 epilepsy‐associated genes. Despite these methodologic and population differences, our study found a similar overall diagnostic yield, which supports the robustness and generalizability of these findings.

The most commonly identified genes in our study are associated with specific familial epilepsy syndromes such as self‐limited neonatal seizures (*KCNQ2*), self‐limited infantile epilepsy (*PRRT2*), GEFS+ (*SCN1A*), and familial focal epilepsy with variable foci (*DEPDC5* and *NPRL3*).^6^ Although the families in our cohort were not diagnosed with specific familial syndromes, it is not surprising that these genes are well represented in a large cohort of patients with a family history of epilepsy. Comparing the distribution of genetic etiologies in our cohort against the broader population of genetic epilepsies revealed that these “familial syndrome” genes are over‐represented in our cohort, whereas genes that are more consistently associated with neurodevelopmental comorbidity and de novo variants (e.g., *STXBP1*) were under‐represented (Figure 2B). On the other hand, many of the genetic etiologies identified only once in our cohort are typically associated with severe neurodevelopmental phenotypes rather than familial epilepsies, which highlights the phenotypic heterogeneity and possibility of inherited pathogenic variants in these conditions.^29^

In most families (79%) the diagnostic variant followed the expected inheritance pattern based on the family history, demonstrating that family history can be helpful for interpreting genetic test results. Segregation is an important component of ACMG variant classification,^15^ but can be challenging to interpret in familial cases. First‐degree relatives share 50% of their variants on average, so a variant can appear to segregate (e.g., detected in an affected parent) by chance alone. In our cohort, only five variants originally classified as variants of uncertain significance but considered clinically diagnostic were inherited from an affected parent or lineage. Even if these cases were considered nondiagnostic, the overall diagnostic yield (94/484, 19.4%) would be very similar, so it is unlikely that over‐interpretation of familial segregation biased our results.

On the other hand, family history can sometimes be misleading. Twenty‐one percent of families had unexpected variant segregation, most often a de novo variant in the proband that did not explain the family history. A previous study of patients with de novo variants causing epilepsy found that 18% had a family history of seizures.^18^ Another study of pediatric genetic epilepsies reported that when causative variants in autosomal dominant genes were inherited from a parent rather than de novo, the transmitting parent was more often unaffected (36 families) than affected (12 families).^18^ Together, these observations convey the complexity of genetic testing in familial epilepsies, where there can be multiple independent etiologies in some families and incomplete penetrance in other families. Of interest, in our cohort probands with affected ancestors in both parental lineages (Group Cin Figure 1) was the familial pattern with the lowest diagnostic yield (2/32 probands, 6%; see Table S4). One possible hypothesis is that these families are more often oligogenic or polygenic, with different genetic influences on each side of the family, rather than monogenic.

Our study relied on dynamic testing strategies that varied between individuals, reflecting real‐world clinical practice. Gene panels were the most commonly performed test, reflecting standard clinical practice during the study period. The standard of care is moving toward exome and genome sequencing as the preferred testing strategy for epilepsy, following professional society guidelines.^6^, ^7^ In our cohort the diagnostic yield was similar for gene panels (16%) and exome sequencing (18%). The selection of tests for each patient reflects a combination of clinical factors and practical concerns such as insurance coverage and cost, which have changed over time and vary between countries. The potential for selection biases should lead to caution when comparing the diagnostic yields of different tests and test combinations. The same is true of all other retrospective observational studies of genetic testing for epilepsy.

Our study had several limitations. First, we examined a retrospective cohort of patients who were referred to an academic neurogenetics clinic, attended the evaluation, and completed genetic testing. Although genetic testing was offered to all eligible probands evaluated in our program, there is likely selection bias in which patients were referred and chose to attend the evaluation. Therefore, our cohort may not be representative of an unselected population of all people with epilepsy, where the frequency of genetic etiologies may potentially differ. Second, dynamic genetic testing strategies reflect real‐world clinical practice, but also introduce potential selection bias that limit the direct comparison of the yield of different tests. For example, gene panels vary in their gene lists and techniques, potentially missing recently identified genes and technically challenging variants (e.g., PRRT2 c.649dupC); copy number variants are not detected by all sequencing tests; and some conditions may require special testing beyond those we included in this study (e.g., methylation studies for Angelman syndrome). Third, we did not systematically collect data on the clinical impacts of genetic diagnoses in our cohort. Identifying a genetic etiology of epilepsy has been show to alter treatment in approximately half of cases,^11^, ^30^ and has many benefits to patients and families beyond immediate management changes.^31^ Even though familial epilepsies are typically milder than the patient cohorts included in these prior studies, we believe that these numbers represent an appropriate benchmark for our cohort, as well.

## 5 CONCLUSION

In conclusion, familial epilepsy has a substantial rate of genetic diagnosis and is an appropriate indication for genetic testing. Familial factors such as the number or pattern of affected relatives did not influence the likelihood of genetic diagnosis, suggesting that all families with epilepsy can be considered for genetic testing. Familial patterns can help interpret genetic test results, while also revealing the complexities of incomplete penetrance and independent epilepsy etiologies in families.

## AUTHOR CONTRIBUTIONS

C.A.E., J.C., I.V., and I.H. contributed to study concept and design and data analysis. All authors contributed to data acquisition and drafting of the manuscript and figures.

## CONFLICT OF INTEREST STATEMENT

None of the authors has any conflict of interest to disclose relevant to this research.

## ETHICS STATEMENT

We confirm that we have read the Journal's position on issues involved in ethical publication and affirm that this report is consistent with those guidelines.

## Supporting information


Data S1.

## Data Availability

The de‐identified data that support the findings of this study are available from the corresponding author upon reasonable request.
