# Supplementary material for: Genetic testing for familial epilepsies: Diagnostic yield and genetic findings
Source: Epilepsia. 2026 Mar 8;67(6):3048–57. doi: 10.1002/epi.70160 (PMC13285230; doi:10.1002/epi.70160)
Supplement: Supplementary file 1 — Data S1. [file EPI-67-3048-s001.pdf]

Genetic testing for familial epilepsies: diagnostic yield and genetic findings

Colin A. Ellis, et al.

SUPPLEMENTAL MATERIAL

Table of Contents

SUPPLEMENTAL TABLE 1. ELIGIBLE PROBANDS WHO DID NOT COMPLETE GENETIC TESTING 2

SUPPLEMENTAL TABLE 2. GENETIC DIAGNOSES 3

SUPPLEMENTAL TABLE 3. COMBINATIONS OF GENETIC TESTS 6

SUPPLEMENTAL TABLE 4. RESULTS OF FAMILIAL PATTERNS AND SEGREGATION ANALYSIS 7

SUPPLEMENTAL TABLE 5: STUDIES REPORTING DIAGNOSTIC YIELD OF GENETIC TESTING IN FAMILIAL EPILEPSIES 8

Supplemental Table 1. Eligible probands who did not complete genetic testing

Characteristic	Genetic testing Completed (n = 484)	Genetic testing Not completed (n = 35)	p-value
Sex female, n (%)	247 (51%)	17 (49%)	0.92[a]
Age at evaluation, median (IQR)	11 (5, 17)	12 (4, 19)	0.64[b]
Age at epilepsy onset, median (IQR)	5 (2, 11)	7 (3, 14)	0.32[b]
Drug resistant, n (%)	124 (26%)	8 (23%)	0.87[a]
Intellectual disability, n (%)	101 (21%)	5 (14%)	0.47[c]
Epilepsy type, n (%)			0.45[c]
Focal	209 (43%)	14 (40%)	
Generalized	138 (29%)	12 (34%)	
DEE	46 (10%)	1 (3%)	
Febrile seizures plus	11 (2%)	1 (3%)	
Unclassified	63 (13%)	7 (20%)	
Brain MRI, n (%)			0.93[c]
Normal or nonspecific	393 (75%)	29 (83%)	
Malformation of cortical development	31 (6%)	2 (6%)	
Other epileptogenic lesion	22 (5%)	1 (3%)	
MRI not performed/not available	68 (14%)	3 (9%)	

[a]Chi-squared test, [b]Mann-Whitney U test, [c]Fisher exact test
Abbreviations: DEE, developmental and epileptic encephalopathy; IQR, interquartile range.

Supplemental Table 2. Genetic diagnoses
Abbreviations: ACMG = American College of Medical Genetics; AD = autosomal dominant; AR = autosomal recessive, CMA = chromosomal microarray del/dup = deletion/duplication, ES = exome sequencing, Hemi. = hemizygous, Het. = heterozygous, Hom. = homozygous, LP = likely pathogenic, P = pathogenic, VUS = variant of uncertain significance.
Note: ACMG classifications were made at the time of diagnosis.

A. Single gene variants

N	Gene	c.	p.	Inheritance	Variant type	Zygosity	Gene MOI	ACMG	Test
11	KCNQ2	c.587C>T	p.Ala196Val	Unknown	Missense	Het.	AD	P	Panel
11	KCNQ2	c.544G>A	p.Val182Met	Unknown	Missense	Het.	AD	LP	Panel
11	KCNQ2	c.916G>A	p.Ala306Thr	Paternal	Missense	Het.	AD	P	Panel
11	KCNQ2	c.285C>A	p.Tyr95*	Maternal	Nonsense	Het.	AD	P	Panel
11	KCNQ2	c.587C>T	p.Ala196Val	Maternal	Missense	Het.	AD	P	Panel
11	KCNQ2	c.644delG	p.Gly215Glufs*58	Maternal	Frameshift	Het.	AD	P	Panel
11	KCNQ2	Exons 2-10 dup		Maternal	Intragenic del/dup	Het.	AD	VUS	Panel
11	KCNQ2	c.1678C>T	p.Arg560Trp	De novo	Missense	Het.	AD	P	Panel
11	KCNQ2	Exons 7-12 dup		Maternal	Intragenic del/dup	Het.	AD	LP	Panel
11	KCNQ2	c.916G>A	p.Ala306Thr	Paternal	Missense	Het.	AD	P	Panel
11	KCNQ2	c.106del	p.Arg36Glyfs*6	Paternal	Frameshift	Het.	AD	P	ES
8	NPRL3	Exon 2 del		Maternal	Intragenic del/dup	Het.	AD	P	Panel
8	NPRL3	Exons 2-9 del		Maternal	Intragenic del/dup	Het.	AD	LP	ES
8	NPRL3	Exons 2-9 del		Maternal	Intragenic del/dup	Het.	AD	P	Panel
8	NPRL3	c.301C>T	p.Gln101*	Paternal	Nonsense	Het.	AD	P	Panel
8	NPRL3	Exons 4-9 dup		Paternal	Intragenic del/dup	Het.	AD	LP	Panel
8	NPRL3	Exons 1-2 del		Paternal	Intragenic del/dup	Het.	AD	P	Panel
8	NPRL3	c.349del	p.Glu117Lysfs*5	Paternal	Frameshift	Het.	AD	P	ES
8	NPRL3	Exons 1-3 del		Paternal	Intragenic del/dup	Het.	AD	P	ES
7	PRRT2	c.649dup	p.Arg217Profs*8	Paternal	Frameshift	Het.	AD	P	Panel
7	PRRT2	c.649dupC	p.Arg217Profs*8	Maternal	Frameshift	Het.	AD	P	ES
7	PRRT2	c.649dupC	p.Arg217Profs*8	Paternal	Frameshift	Het.	AD	P	Panel
7	PRRT2	c.649dupC	p.Arg217Profs*8	De novo	Frameshift	Het.	AD	P	Panel
7	PRRT2	c.649dup	p.Arg217Profs*8	Paternal	Frameshift	Het.	AD	P	Panel
7	PRRT2	c.649delC	p.Arg217Glufs*12	Maternal	Frameshift	Het.	AD	P	Panel
7	PRRT2	c.649delC	p.Arg217Glufs*12	Maternal	Frameshift	Het.	AD	P	Panel
7	SCN1A	c.5414T>C	p.Phe1805Ser	Maternal	Missense	Het.	AD	VUS	Panel
7	SCN1A	c.413T>G	p.Ile138Ser	Maternal	Missense	Het.	AD	VUS	Panel
7	SCN1A	c.1199T>C	p.Met400Thr	Paternal	Missense	Het.	AD	VUS	Panel
7	SCN1A	c.5179G>C	p.Asp1727His	Maternal	Missense	Het.	AD	LP	Panel
7	SCN1A	c.413T>G	p.Ile138Ser	Maternal	Missense	Het.	AD	LP	Panel
7	SCN1A	c.2624C>T	p.Thr875Met	Maternal	Missense	Het.	AD	P	Panel

7	SCN1A	c.413T>G	p.Ile138Ser	Unknown	Missense	Het.	AD	LP	Panel
5	DEPDC5	c.982C>T	p.Arg328*	Maternal	Nonsense	Het.	AD	P	Panel
5	DEPDC5	c.3802C>T	p.Arg1268*	Maternal	Nonsense	Het.	AD	P	ES
5	DEPDC5	Exon 41 del		Paternal	Intragenic del/dup	Het.	AD	P	ES
5	DEPDC5	c.562+1G>A	p.?	Maternal	Splice	Het.	AD	LP	ES
5	DEPDC5	c.947-13_1008del	p.?	Maternal	Splice	Het.	AD	LP	ES
4	SCN2A	c.1314G>C	p.Glu438Asp	Maternal	Missense	Het.	AD	VUS	Panel
4	SCN2A	c.4898T>C	p.Ile1633Thr	Maternal	Missense	Het.	AD	VUS	Panel
4	SCN2A	c.4434A>C	p.Gln1478His	Maternal	Missense	Het.	AD	LP	Panel
4	SCN2A	c.2872A>G	p.Met958Val	De novo	Missense	Het.	AD	VUS	Panel
3	KRIT1	c.2026-2A>G	p.?	Paternal	Splice	Het.	AD	LP	Panel
3	KRIT1	c.875_876del	p.Leu292Profs*6	Unknown	Frameshift	Het.	AD	P	Panel
3	KRIT1	c.1563+1G>A	p.?	Unknown	Splice	Het.	AD	P	Panel
3	SLC6A1	c.889G>A	p.Gly297Arg	De novo	Missense	Het.	AD	LP	Panel
3	SLC6A1	c.373G>A	p.Val125Met	Maternal	Missense	Het.	AD	P	Panel
3	SLC6A1	c.1084G>C	p.Gly362Arg	Maternal mosaic	Missense	Het.	AD	P	ES
3	SYNGAP1	Whole gene dup		De novo	Intragenic del/dup	Het.	AD	VUS	Panel
3	SYNGAP1	c.3557C>T	p.Ser1186Leu	De novo	Missense	Het.	AD	LP	Panel
3	SYNGAP1	5'UTR_EX3del		Maternal mosaic	Intragenic del/dup	Het.	AD	P	ES
1	ANKRD11	c.2751_2752delTG	p.Lys919Glufs*6	De novo	Frameshift	Het.	AD	P	ES
1	ARFGEF1	c.4627C>T	p.Arg1543*	Maternal	Nonsense	Het.	AD	P	GS
1	CACNA1A	c.4198A>T	p.Lys1400*	Maternal	Nonsense	Het.	AD	P	ES
1	CHD2	c.3520_3528del	p.Gly1174_Leu 1176del	Paternal	deletion	Het.	AD	LP	ES
1	FGFR3	c.749C>G	p.Pro250Arg	De novo	Missense	Het.	AD	P	ES
1	GABRB2	c.745A>G	p.Thr249Ala	De novo	Missense	Het.	AD	VUS	Panel
1	GABRG2	c.570C>A	p.Cys190*	Maternal	Nonsense	Het.	AD	P	Panel
1	KANSL1	c.659_660del	p.Thr220Asnfs*4	Unknown	Frameshift	Het.	AD	LP	ES
1	KCNA1	c.1214C>T	p.Pro405Leu	De novo	Missense	Het.	AD	VUS	Panel
1	KCNA6	c.1126C>G	p.Leu376Val	De novo	Missense	Het.	AD	VUS	ES
1	KIF1A	c.31C>T	p.Arg11Trp	De novo	Missense	Het.	AD	P	Panel
1	LGI1	c.504-9_505del		Maternal	Intragenic del/dup	Het.	AD	LP	ES
1	MYCBP2	c.3140_3141dup	p.Gly1048Metfs*8	De novo	Frameshift	Het.	AD	LP	ES
1	NCKAP1	c.1022+1G>A	p.?	Maternal	Splice	Het.	AD	LP	ES
1	PTPN11	c.330A>C	p.Glu110Asp	Paternal	Missense	Het.	AD	LP	ES
1	PUF60	c.649 C>T	p.Gln217*	Unknown	Nonsense	Het.	AD	P	ES
1	RORA	c.920-2A>G	p.?	Maternal	Splice	Het.	AD	P	ES
1	SCN8A	c.3563G>A	p.Arg1188Gln	Maternal	Missense	Het.	AD	LP	Panel
1	SGCE	c.619del	p.Arg207Glyfs*12	Maternal	Frameshift	Het.	AD	P	Panel
1	SLC2A1	c.259G>A	p.Val87Ile	Unknown	Missense	Het.	AD	P	Panel
1	TSC1	c.1525C>T	p.Arg509*	Paternal	Nonsense	Het.	AD	P	Panel
1	TSC2	c.1864C>T	p.Arg622Trp	Paternal	Missense	Het.	AD	P	Panel
1	WDFY3	c.2785C>T	p.Arg929*	Maternal	Nonsense	Het.	AD	P	ES
1	MT-CYB	m.15183T>C	p.Ile145Thr	Maternal	Missense	Het.	Mito	LP	ES

1	MT-TK	m.8344A>G	p.?	Maternal	Missense	Het.	Mito	P	ES
1	GRIA3	c.2497G>A	p.Gly833Arg	Unknown	Missense	Hemi.	XL	P	ES
1	MECP2	c.709_710del	p.Gly237Trpfs*21	De novo	Frameshift	Hemi.	XL	P	Panel
1	NEXMIF	c.617dup	p.Phe207Leufs*5	Maternal	Frameshift	Hemi.	XL	P	Panel
1	CLN6	c.13C>T;<br>c.843G>C	p.Arg5Trp;<br>p.Trp281Cys	Biallelic	Missense	Comp<br>Het.	AR	LP; LP	ES
1	MED23	c.4006C>T;<br>c.3656A>G	p.Arg1336*;<br>p.H1219R	Biallelic	Nonsense;<br>Missense	Comp<br>Het.	AR	P; LP	ES
1	NRROS	c.1222A>C	p.Asn408His	Biallelic	Missense	Hom.	AR	VUS	GS
1	PNPT1	c.223-1G>A ;<br>c.1520C>G	p.Ala507Gly;<br>p.?	Biallelic	Missense;<br>Splice	Comp<br>Het.	AR	LP; LP	ES
1	TBC1D24	c.946_947delAT;<br>c.679C>T	p.Ile316Hfs*11;<br>p.Arg227Trp	Biallelic	Frameshift;<br>Missense	Comp<br>Het.	AR	P; LP	ES
1	VPS13A	c.6358A>T;<br>9q21 del	p.Lys2120*;<br>506 kb del	Biallelic	Nonsens;<br>Deletion	Comp<br>Het.	AR	P; P	ES

B. Copy number variants

N	Locus	size	Inheritance	Zygosity	ACMG	Test
3	16p11.2 del	542 kb	Maternal	Het.	P	Panel
3	16p11.2 del	513 kb	Paternal	Het.	P	Panel
3	16p11.2 del	612 kb	Unknown	Het.	P	CMA
2	15q13.3 del	1.8 Mb	Unknown	Het.	P	CMA
2	15q13.3 del	2.0 Mb	Unknown	Het.	LP	CMA
1	15q11.2 del	511 kb	Unknown	Het.	P	CMA
1	15q11.2 dup	4.9 Mb	Unknown	Het.	P	Panel
1	16p11.2 dup	450 kb	Unknown	Het.	P	CMA
1	16p13.11 del	1.4 Mb	Unknown	Het.	P	CMA
1	20p13 del;<br>20q13 dup	1.2 Mb;<br>4.7 Mb	De novo	Het.	P	ES
1	2p16.3 del	144 kb	Maternal	Het.	P	CMA
1	9q33.1 del	169 kb	Maternal	Het.	LP	CMA

C. Other variants

N	Gene or variant	Inheritance	Zygosity	ACMG	Test
1	Trisomy X	Unknown	Trisomy	P	CMA
1	FMR1 >200 repeats	Maternal	Het. Mosaic	P	Fragile X

Abbreviations: ACMG = American College of Medical Genetics variant classification system; AD = autosomal dominant; AR = autosomal recessive; CMA = chromosomal microarray; ES = exome sequencing; GS = genome sequencing; Het. = heterozygous; Comp. Het. = compound heterozygous; MOI = mode of inheritance; LP = likely pathogenic; P = pathogenic; VUS = variant of uncertain significance; XL = X-linked.

Supplemental Table 3. Combinations of Genetic Tests

Tests	Performed (N)	Diagnostic (N)	Yield (%)
One test			
CMA	15	3	20%
Panel	232	46	20%
ES/GS	118	27	23%
Two tests			
CMA + ES/GS	25	7	28%
CMA + Panel	43	11	26%
Panel + ES/GS	33	4	12%
Three tests			
CMA + Panel + ES/GS	18	1	6%
**Total**	**484**	**99**	**20%**

Supplemental Table 4. Results of familial patterns and segregation analysis

				Variant segregation		
**Familial Pattern**	**Families (N)**	**Diagnostic (N)**	**Yield (%)**	**Expected**	**Unexpected**	**Unknown[a]**
Parent affected	261	60	23%	40	8	12
One lineage	105	20	19%	14	4	2
Both lineages	32	2	6%	1	0	1
Siblings only	77	16	21%	9	4	3
Children only	9	1	11%	0	1	0
**Total**	**484**	**99**	**20%**	**64**	**17**	**18**

[a]Parents were unavailable for testing

Supplemental Table 5: Studies reporting diagnostic yield of genetic testing in familial epilepsies

Study (ref)	Country	Population	FH definition	Test	Yield, n (%)
d'Orsi et al 2017 ([19])	Italy	Epilepsy + ID	NR	CMA	6/20 (30%)
Perucca 2017 ([20])	Australia	MRI-negative focal epilepsy	1st or 2nd degree	ES	5/40 (13%)
Liu 2018 ([31])	China	Pediatric refractory epilepsy	NR	Panel (153 genes)	7/27 (26%)
Tsai 2018 ([21])	Taiwan, Malaysia	Focal epilepsy	1st or 2nd degree	Panel (21 genes)	4/99 (4%)
Balciuniene 2019 ([22])	USA	Childhood epilepsy	NR	ES	7/50 (14%)
Costain 2019 ([23])	Canada	Childhood epilepsy	1st degree	ES	5/16 (31%)
Alsubaie 2020 ([24])	Saudi Arabia	Epilepsy	NR	ES	4/21 (19%)
Fortin 2020 ([25])	Canada	GEFS+	Any FS or FS+	CMA	4/12 (33%)
Krenn 2020 ([26])	Austria	Non-acquired focal epilepsy	One 1st or two 2nd degree	ES	6/37 (16%)
Licchetta 2020 ([27])	Italy	Sleep-related hypermotor epilepsy	1st or 2nd degree	ES	3/16 (19%)
de Sainte Agathe 2025 ([18])	France	Focal epilepsy	NR	Panel (68 genes)	21/89 (24%)
Ellis 2025 (current study)	USA	Epilepsy	One 1st or two 2nd-3rd degree	Dynamic	99/484 (20%)

Abbreviations: CMA = chromosomal microarray, ES = exome sequencing, FH = family history, FS = febrile seizures, FS+ = febrile seizures-plus, GEFS+ = genetic epilepsy with febrile seizures plus, ID = intellectual disability, NR = not reported
